# ANKRD22 promotes progression of non-small cell lung cancer through transcriptional up-regulation of E2F1

**DOI:** 10.1038/s41598-017-04818-y

**Published:** 2017-06-30

**Authors:** Jun Yin, Wenfan Fu, Lu Dai, Zeyong Jiang, Hongying Liao, Wenbin Chen, Lei Pan, Jian Zhao

**Affiliations:** Department of Chest Surgery, Affiliated Cancer Hospital & Institute of Guangzhou Medical University, Guangzhou, 510095 Guangdong China

## Abstract

Lung cancer is the leading cause of death among all malignancies due to rapid tumor progression and relapse; however, the underlying molecular mechanisms of tumor progression are unclear. In the present study, we identified ANKRD22 as a novel tumor-associated gene in non-small cell lung cancer (NSCLC). According to the clinical correlation analysis, ANKRD22 was highly expressed in primary cancerous tissue compared with adjacent cancerous tissue, and high expression levels of ANKRD22 were significantly correlated with relapse and short overall survival time. Knockdown and overexpression analysis revealed that ANKRD22 promoted tumor progression by increasing cell proliferation. In xenograft assays, knockdown of ANKRD22 or *in vivo* treatment with ANKRD22 siRNA inhibited tumor growth. Furthermore, ANKRD22 was shown to participate in the transcriptional regulation of E2F1, and ANKRD22 promoted cell proliferation by up-regulating the expression of E2F1 which enhanced cell cycle progression. Therefore, our studies indicated that ANKRD22 up-regulated the transcription of E2F1 and promoted the progression of NSCLC by enhancing cell proliferation. These findings suggest that ANKRD22 could potentially act as a novel therapeutic target for NSCLC.

## Introduction

Lung cancer has the highest morbidity and mortality of all cancers worldwide, and approximately 80% of cases are non-small cell lung cancer (NSCLC)^1^. Due to growing tobacco consumption and serious environmental pollution, the incidence of NSCLC is increasing annually^2^. However, most NSCLC patients have progressed when diagnosed as specific symptoms and signs are lacking in the early stage of tumor onset, and more than 90% of treatment failure and mortality is due to extensive metastasis and recurrence^3^.

It has been demonstrated that genetic alterations are key events in the tumorigenesis of many malignant tumors, including NSCLC^4^. Tumor-associated genetic alterations usually result in activation of oncogenes and inactivation of tumor suppressor genes, and many oncogenes and tumor suppressor genes have been determined and used for molecular targeted therapy of NSCLC^5–7^. Although important clinical advances have been achieved, the molecular mechanisms of NSCLC, especially the mechanism involved in tumor progression, are still unclear, and treatments for tumor metastasis and recurrence are still lacking^8^. Therefore, more studies are needed to further identify novel key genes which would contribute to understanding tumor progression and identifying a better way to control tumor metastasis and recurrence in NSCLC, although this is still at the exploratory stage^9^.

Usually, a succession of genetic and epigenetic events, which occur during tumor progression, can result in discrete changes in transcriptional regulation, leading to progressive aberrant gene expression that supports tumor progression^10^. Therefore, in order to determine the mechanism underlying the regulation of NSCLC progression, we performed gene expression profiling analysis in adjacent, primary and metastatic carcinoma tissues obtained from the same NSCLC patient. From the results, we identified ANKRD22 (ankyrin repeat domain 22) to be successively up-regulated in adjacent, primary and metastatic carcinoma tissues, and significantly affected tumor growth as a novel tumor-associated gene in NSCLC. In addition, high expression levels of ANKRD22 were significantly associated with relapse and short overall survival time in NSCLC patients. ANKRD22 is an ankyrin repeat protein with four copies of the ankyrin motif. As one of the most common protein motifs in nature, the ankyrin motif is a canonical helix-loop-helix-β-hairpin/loop fold approximately 30–34 residues in length, and the diversity of unrelated molecules with which the ankyrin motif interacts is reflected in many cellular processes, including transcriptional regulation, signal transduction, development, inflammatory response, cell cycle regulation, apoptosis, and oncogenesis^11–13^. Furthermore, we investigated the biological functions of ANKRD22 in NSCLC and found that ANKRD22 affected cell proliferation and tumorigenicity in nude mice. Remarkably, direct injection of ANKRD22 small interfering RNA (siRNA) into xenograft tumors inhibited tumor growth. Mechanistically, we found that ANKRD22 affected cell proliferation and the cell cycle by transcriptional up-regulation of E2F1, and the correlation between ANKRD22 and E2F1 expression was significantly positive in NSCLC tissues. E2F1 is a transcription factor associated with cell cycle regulation, and recent evidence has shown that aberrant expression of E2F1 in cancers is relevant for cancer progression^14, 15^. Therefore, these results not only shed light on the mechanisms of NSCLC progression, but have also led to the discovery of a new biomarker and therapy target for tumor metastasis and recurrence of NSCLC.

## Results

### Screening and identification of ANKRD22 as a novel tumor-associated gene in NSCLC progression

**Figure 1:**
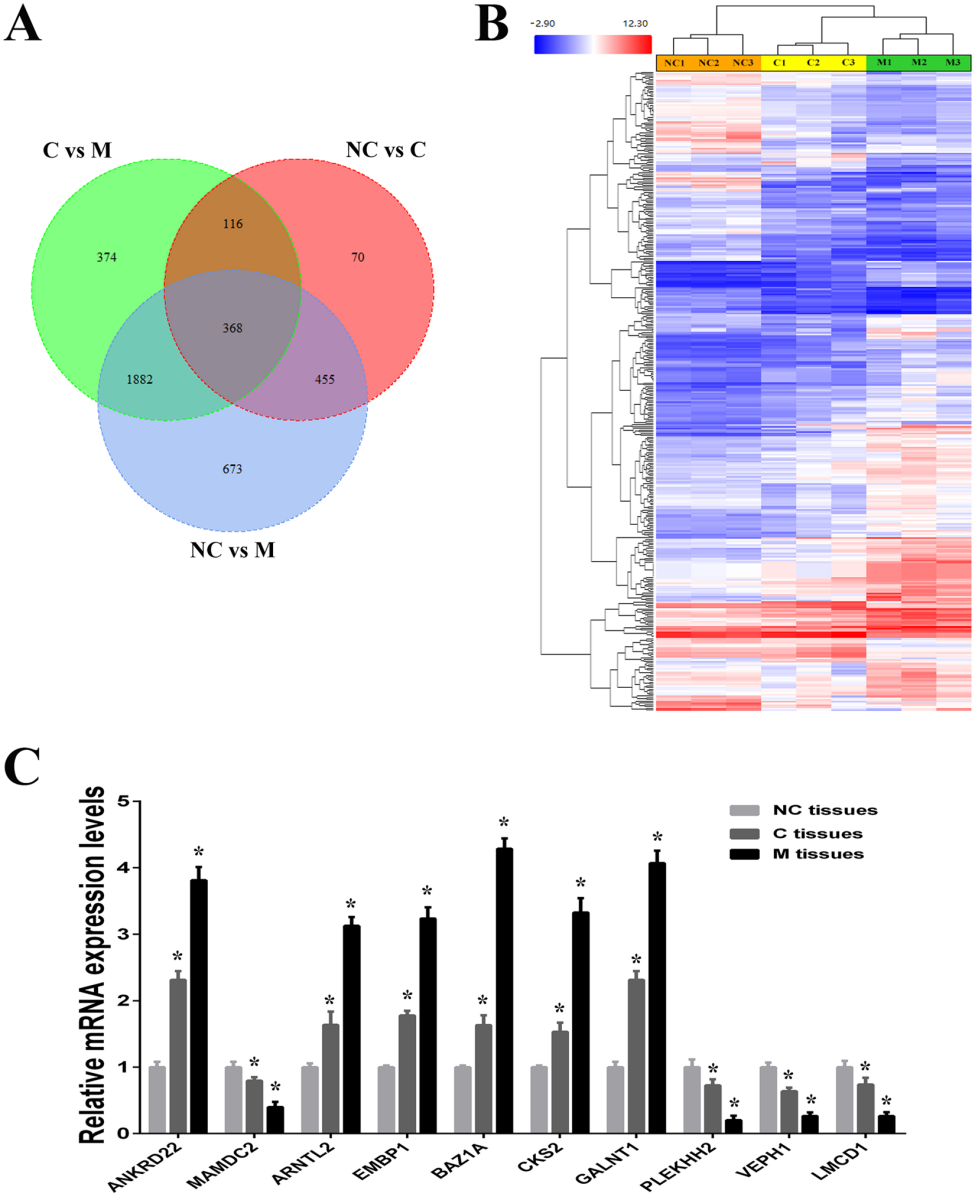
Gene expression profile analysis of NC, C and M tissues in NSCLC. (**A**) Venn diagram showing the overlap in differentially expressed genes in three comparisons between NC, C and M tissues. (**B**) Clustering of successively changed genes in NC, C and M tissues. Color corresponds to the expression levels of genes according to the color bar scale. (**C**) Expression levels of 10 candidate genes in 3 pairs of NSCLC samples (NC, C and M tissues) were measured by RT-PCR. NC, adjacent carcinoma tissues; C, primary carcinoma tissues; M, metastatic carcinoma tissues. (n = 3, *P < 0.05).

**Figure 2:**
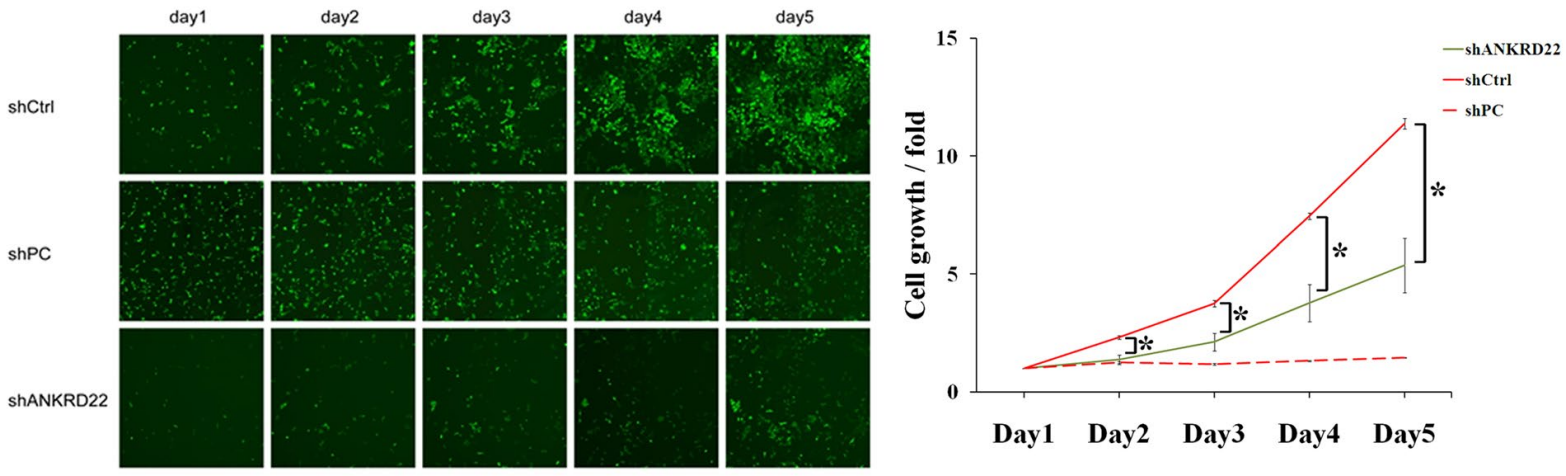
Effect of ANKRD22 on cell growth. H1299 cells transfected with ANKRD22-shRNA (shANKRD22), negative control shRNA (shCtrl) and positive control shRNA (shPC) were observed by High Content Imaging Pathway Celigo for 5 days. The cell growth curve (fold change) indicated that knockdown of ANKRD22 expression significantly inhibited H1299 cell growth. (n = 3, *P < 0.05).

We performed RNA expression profiling in three tissue samples (adjacent, primary and metastatic carcinoma tissues; NC, C and M tissues) obtained from patients with NSCLC. Patient characteristics are shown in Table S1. Using comparative analysis, the mRNA expression of 368 genes was statistically different in NC, C and M tissues (fold change >1.5 or <−1.5, *P* < 0.05) (Fig. 1A,B). Moreover, 271 genes were successively up-regulated or down-regulated in C and M tissues compared with NC tissues. Ten candidate genes, which were confirmed as successively changed genes in NC, C and M tissues using RT-PCR, were selected to screen novel regulatory genes related to tumor progression in NSCLC (Fig. 1C). Using high content screening (HCS), cell growth was detected for five days after transfection of candidate genes shRNAs, negative control shRNA and positive control shRNA, respectively (Table S2, Figs 2 and S1). Remarkably, of these 10 candidate genes, only suppression of ANKRD22 significantly inhibited H1299 cell growth (an average of 2.12-fold inhibition) compared with negative control shRNA. Therefore, we screened out the novel tumor-associated gene, ANKRD22, which was successively up-regulated during tumor progression and influenced tumor cell growth in NSCLC.

### Associations between ANKRD22 and the clinical characteristics of NSCLC

**Figure 3:**
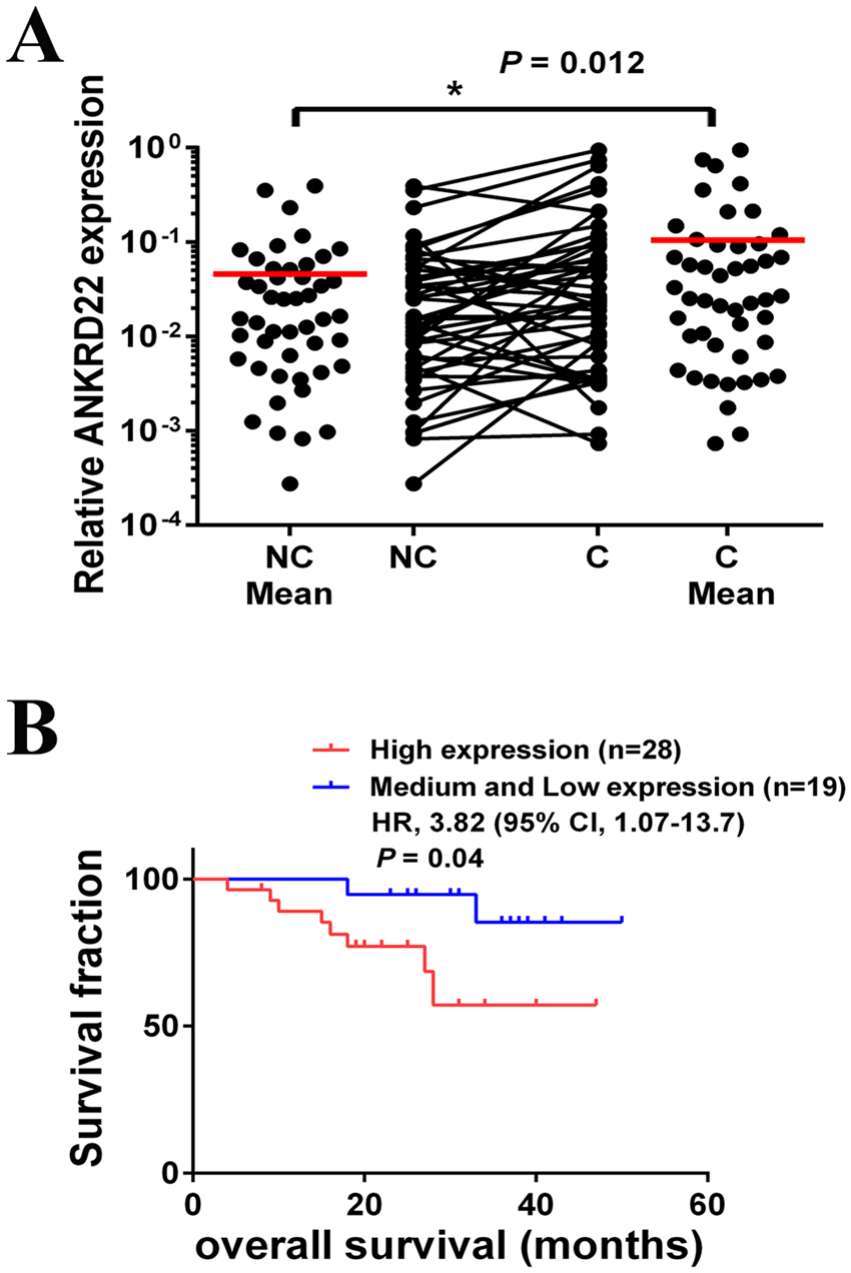
ANKRD22 expression levels were associated with poor prognosis of NSCLC. (**A**) Scatter plots of expression levels of ANKRD22 in NC and C tissues. NC, adjacent carcinoma tissues; C, primary carcinoma tissues. (**B**) Kaplan-Meier analysis of overall survival in NSCLC patients with primary tumors assessed for expression levels of ANKRD22. The *P* values correspond to hazard ratios (HR). (n = 3, *P < 0.05).

In order to analyze the clinical correlations of ANKRD22 in NSCLC, the expression status of ANKRD22 in 47 pairs of adjacent and primary NSCLC samples was assessed by RT-PCR. As shown in Fig. 3A, the expression of ANKRD22 in primary tissues (C tissues) was significantly higher than that in adjacent tissues (NC tissues). In order to investigate whether the expression of ANKRD22 was correlated with the clinicopathologic features of NSCLC patients, the samples were divided into three groups depending on the relative expression of ANKRD22 in C tissues compared with NC tissues: High (fold change ≥1.5, n = 28), Medium (1.5> fold change ≥1, n = 9) and Low (fold change <1, n = 10). As shown in Table S3, compared with the Medium and Low groups combined, NSCLC patients in the High group were more likely to have a relapse (*P* = 0.03) and metastasis (*P* = 0.078), although the clinical correlation between ANKRD22 and metastasis was not significant. The expression of ANKRD22 was not significantly different by histological type, gender, age, smoking history and TNM stage. Moreover, Kaplan-Meier curves demonstrated that the expression of ANKRD22 was prognostic over the follow-up period, and the difference between the High group and the Medium and Low groups combined in terms of median overall survival (OS, 25 vs. 33 months) was statistically significant (*P* < 0.05) (Fig. 3B). Therefore, aberrant high expression of ANKRD22 was significantly associated with increased tumor progression and shortened OS time in NSCLC.

### Promotion of cell proliferation *in vitro* by ANKRD22

**Figure 4:**
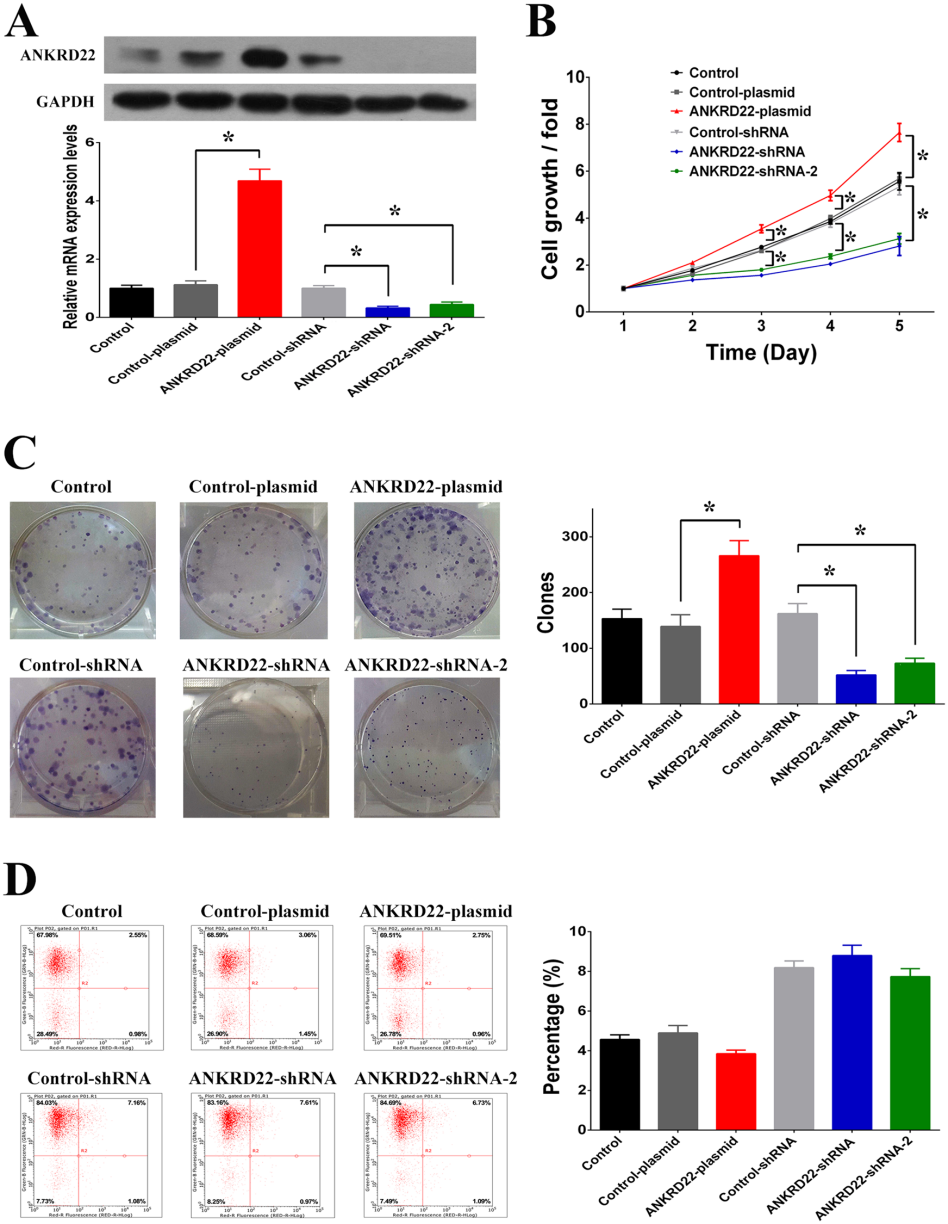
ANKRD22 regulated cell proliferation. (**A**) The mRNA and protein levels of ANKRD22 were increased after transfection with ANKRD22-plasmid and decreased after transfection with ANKRD22-shRNA in H1299 cells. Full-length blots are presented in Supplementary Figure S6. (**B**) Overexpression of ANKRD22 increased cell growth and knockdown of ANKRD22 decreased cell growth in H1299 cells. (**C**) Overexpression of ANKRD22 increased colony formation capacity and knockdown of ANKRD22 decreased colony formation capacity in H1299 cells. (**D**) Overexpression or knockdown of ANKRD22 had no effect on cell apoptosis in H1299 cells. (n = 3, *P < 0.05).

To investigate the roles of ANKRD22 in NSCLC cells, we used cDNA plasmid or shRNA to enhance and silence the expression of ANKRD22 in H1299 cells (Fig. 4A). Firstly, we analyzed the effect of ANKRD22 on cell growth of NSCLC cells. Following transfection with ANKRD22-plasmid or ANKRD22-shRNA for five days, overexpression of ANKRD22 increased and silencing of ANKRD22 prevented cell growth compared to the negative controls (Fig. 4B). However, it is not known whether ANKRD22 affected cell growth by regulating cell proliferation or cell apoptosis. According to the results of the colony formation assay, overexpression of ANKRD22 promoted and silencing of ANKRD22 inhibited colony formation capacity compared to the negative controls (Fig. 4C). However, overexpression or silencing of ANKRD22 did not affect cell apoptosis compared to the negative controls (Fig. 4D). By using another NSCLC cell line A549, the promotion effects of ANKRD22 on cell proliferation were also observed (Fig. S2). Therefore, these results suggested that up-regulation of ANKRD22 promoted cell growth of NSCLC cells by promoting cell proliferation.

### Promotion of xenograft tumor proliferation *in vivo* by ANKRD22

**Figure 5:**
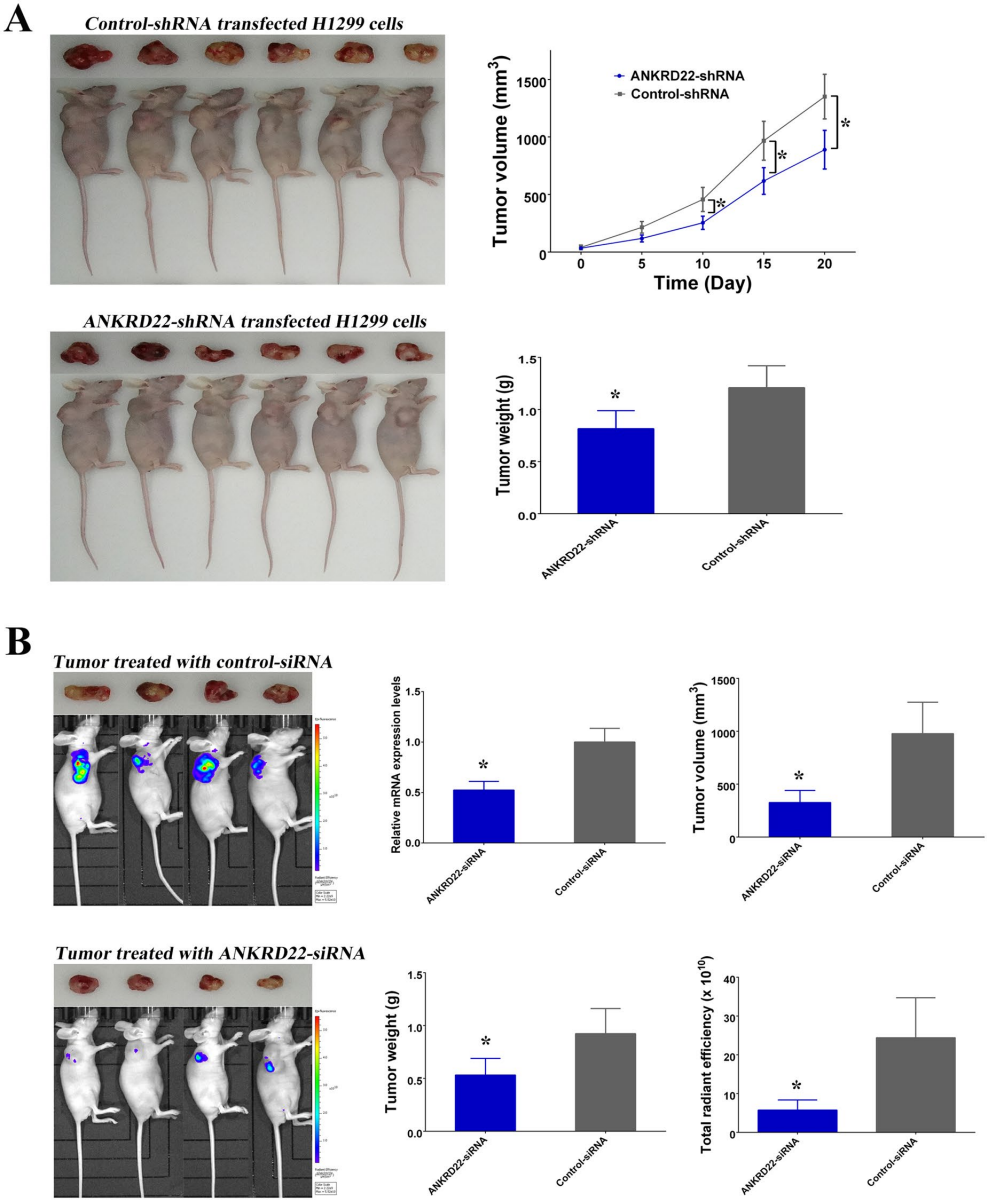
ANKRD22 influenced the growth of xenograft tumor. (**A**) Knockdown of ANKRD22 in H1299 cells by transfection with ANKRD22-shRNA decreased the volumes and weights of xenograft tumors. (**B**) Direct injection of ANKRD22-siRNA inhibited the growth of xenograft tumor *in vivo*. (*P < 0.05).

To confirm tumor promotion by ANKRD22, ANKRD22-shRNA and control-shRNA transfected H1299 cells were injected into nude mice to establish a xenograft model of tumorigenesis. The results showed that ANKRD22-shRNA induced silencing of ANKRD22 resulting in a significant decrease in tumor volumes and weights compared with control-shRNA (Fig. 5A). Furthermore, in order to examine the effects of RNAi silencing ANKRD22 gene on the tumor inhibition, mixture of ANKRD22-siRNA and atelocollagen was directly injected into luciferase labeled xenograft tumors with approximately 10 mm in diameter. After 10 days of treatment, ANKRD22-siRNA treated group clearly decreased ANKRD22 mRNA levels and significantly reduced the volumes, weights and fluorescence intensity of the tumors compared with control-siRNA treated group (Fig. 5B). These results suggested that ANKRD22 also promoted tumor growth *in vivo* and acted as a potential cancer therapy target.

### ANKRD22 is involved in transcriptional regulation of E2F1

**Figure 6:**
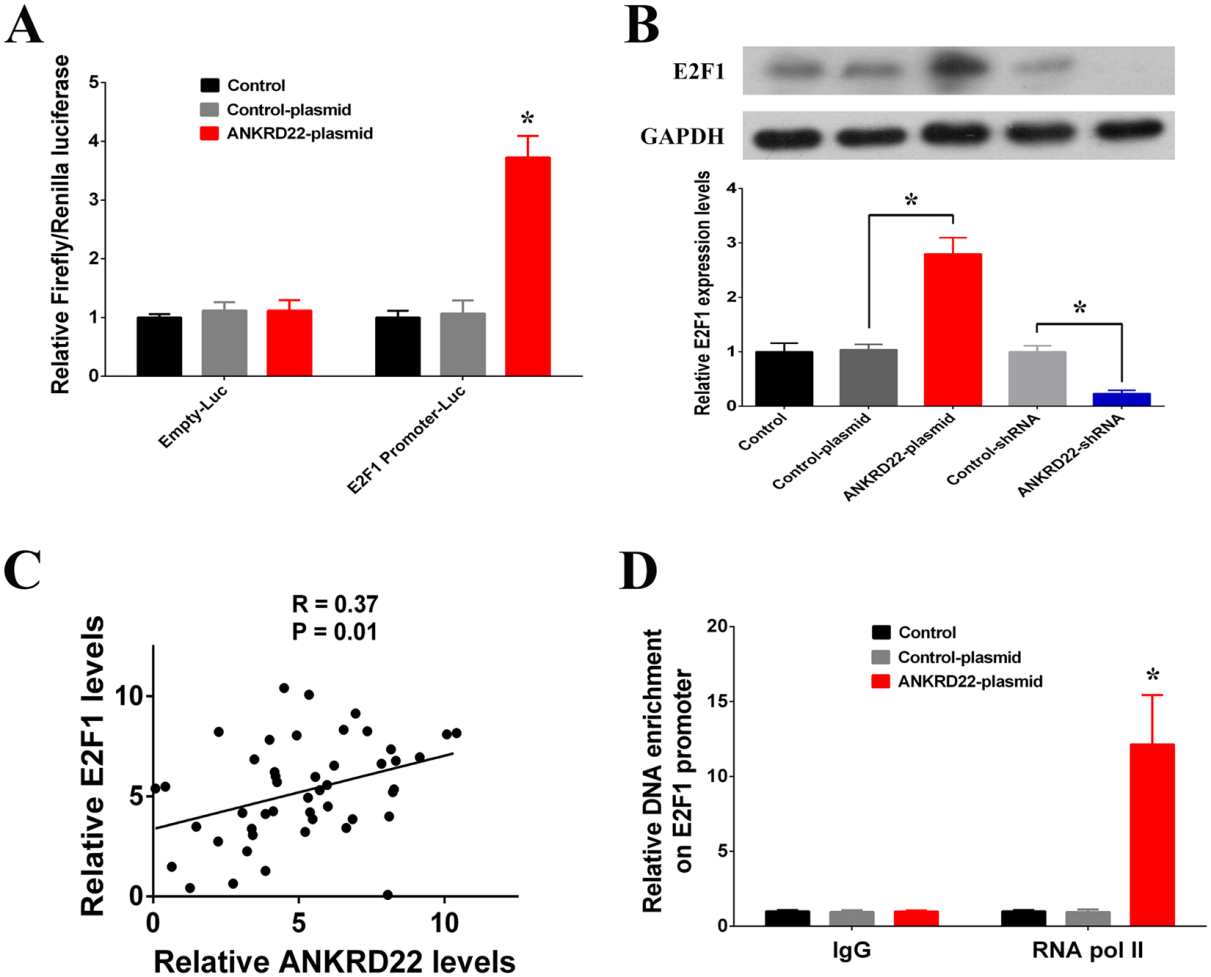
ANKRD22 promoted the expression of E2F1 gene. (**A**) The transcriptional activation of E2F1 by ANKRD22 was detected by luciferase assays. (**B**) Overexpression of ANKRD22 increased and knockdown of ANKRD22 decreased the mRNA and protein expression levels of E2F1 in H1299 cells. Full-length blots are presented in Supplementary Figure S7. (**C**) Correlation between the relative expressions of E2F1 and ANKRD22 in specimens from NSCLC patients expressed using linear regression (solid line). (**D**) RNA polymerase II (RNA Pol II) signal in the E2F1 promoter region was detected by chromatin immunoprecipitation (ChIP) assay. (n = 3, *P < 0.05).

To investigate the regulatory function of ANKRD22 in NSCLC, we used microarray to measure changes in gene expression profiles in response to knockdown of ANKRD22. The results showed that 2530 mRNAs were increased or decreased with more than a 1.5-fold change and *P* < 0.05 in the shRNA-ANKRD22 group compared with the control-shRNA group (Fig. S3). Signal pathway enrichment analysis showed that five pathways in the top ten genes were associated with the cell cycle, suggesting that knockdown of ANKRD22 affected the cell cycle pathway (Tables S4 and S5). Furthermore, of the genes which were associated with these five cell cycle pathways, 44 of 56 were down-regulated in the shRNA-ANKRD22 group. According to the cis-regulatory modules enrichment analysis, most (25 of 44) of these down-regulated cell cycle pathway-associated genes can potentially be bound and regulated by the transcription factor E2F1, which was required for cell cycle progression and was down-regulated in the shRNA-ANKRD22 group. The results of the dual-luciferase reporter assay demonstrated that overexpression of ANKRD22 enhanced the transcriptional activity of the E2F1 gene promoter (Fig. 6A). Moreover, it was confirmed that E2F1 mRNA and protein were up-regulated by overexpression of ANKRD22 and down-regulated by silencing of ANKRD22 (Fig. 6B). In addition, a significant positive correlation was observed between the expression of ANKRD22 and E2F1 which was also significantly upregulated in C tissues compared with NC tissues (Figs 6C and S4). It is well known that RNA polymerase II (RNA pol II) on the gene promoter affects transcriptional activation. Therefore, according to the results of chromatin immunoprecipitation assays, overexpression of ANKRD22 enriched RNA pol II in the E2F1 promoter region suggested that ANKRD22 activated transcriptional activity of the E2F1 promoter and increased the transcription of E2F1 (Fig. 6D). Therefore, these results indicated that ANKRD22 was involved in the transcriptional regulation of E2F1.

### ANKRD22 promoted cell cycle progression by up-regulating E2F1

**Figure 7:**
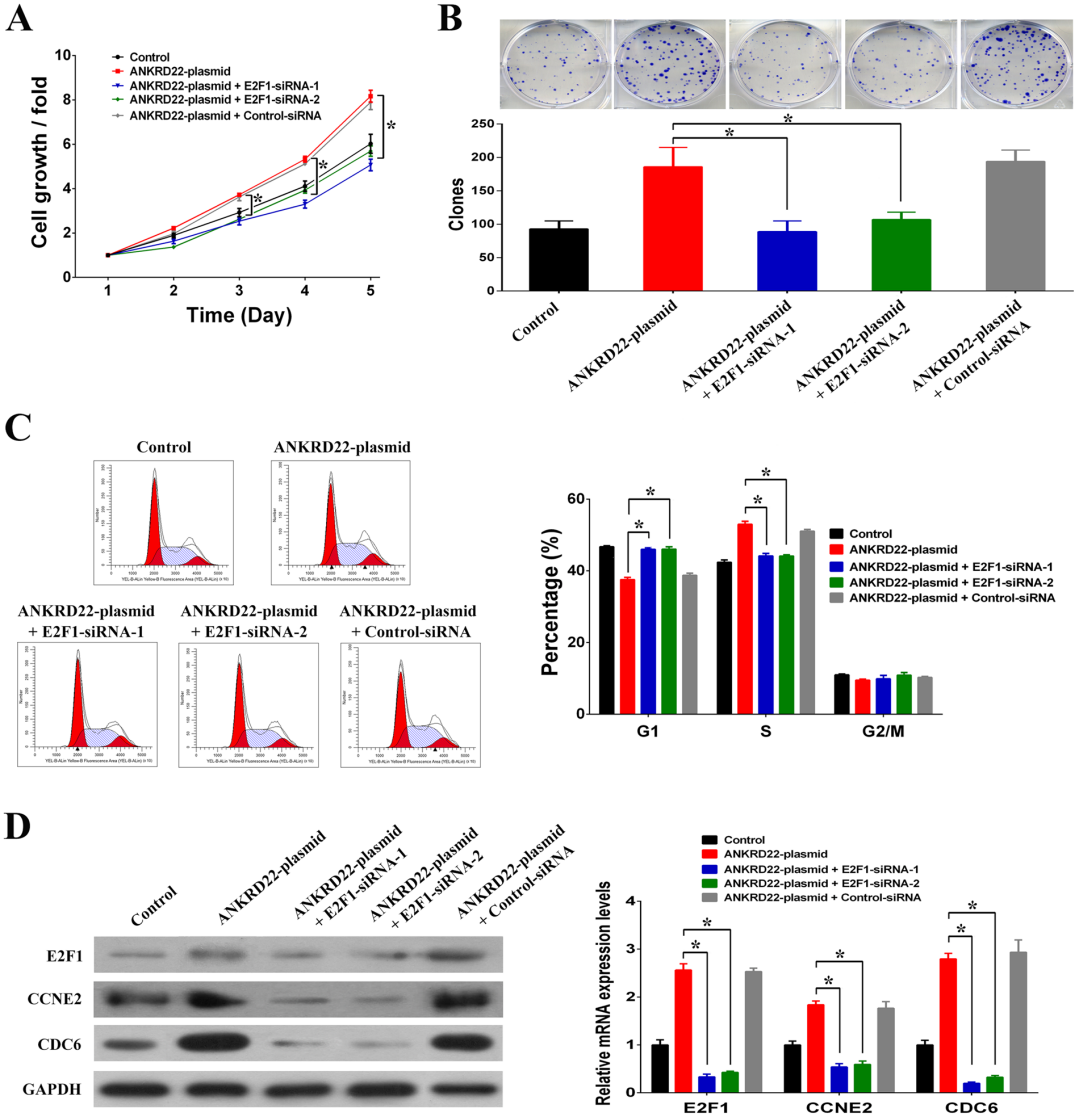
ANKRD22 regulated the cell cycle and cell proliferation by regulating E2F1. (**A**) Silencing of E2F1 reduced the promotion of cell growth by ANKRD22 in H1299 cells. (**B**) Silencing of E2F1 reduced the promotion of colony formation capacity by ANKRD22 in H1299 cells. (**C**) Silencing of E2F1 reduced promotion of the cell cycle by ANKRD22 in H1299 cells. (**D**) Silencing of E2F1 reduced promotion of the expression of E2F1 target genes CCNE2 and CDC6 by ANKRD22. Full-length blots are presented in Supplementary Figure S8. (n = 3, *P < 0.05).

In order to determine whether ANKRD22 regulated cell proliferation through E2F1, we silenced E2F1 with two different short interfering RNAs (siRNAs) in ANKRD22-plasmid transfected H1299 cells. The results showed that knockdown of E2F1 reduced the promotion of cell growth and cell proliferation by ANKRD22 (Fig. 7A,B). As it has been reported that E2F1 is involved in the regulation of cell cycle progression^16^, we further investigated whether ANKRD22 influenced the cell cycle by regulating E2F1. Compared to the negative controls, overexpression of ANKRD22 resulted in an increase in G1-phase of 9.2% and a decrease in S-phase of 10.6%. However, after knockdown of E2F1 in ANKRD22-plasmid transfected H1299 cells, the rate of G1-phase decreased by 7.9% and S-phase increased by 8.7% (Fig. 7C). Cell cycle assays indicated that knockdown of E2F1 inhibited the promotion of cell cycle progression by ANKRD22. In addition, CCNE2 and CDC6, which are E2F1 target genes associated with cell cycle regulation, were up-regulated by overexpression of ANKRD22 and were restored after siRNA-induced silencing of E2F1 (Fig. 7D). By using another NSCLC cell line A549, the regulation effects of ANKRD22 on cell cycle were also observed (Fig. S5). These results revealed that the roles of ANKRD22 in tumor progression were achieved through the transcriptional up-regulation of E2F1, which resulted in the promotion of cell cycle progression.

## Discussion

ANKRD22, which is located within 10q23.31, is an ankyrin repeat protein; however, limited information is available on ANKRD22^17–21^. It has been reported that the expression of ANKRD22 in peripheral blood samples can be used for the diagnosis of pancreatic ductal adenocarcinoma^20^. The present study shows, for the first time, that there is an association between the up-regulation of ANKRD22 and tumor progression in NSCLC. Successive up-regulation of ANKRD22 was observed in adjacent, primary and metastatic carcinoma tissues in the same NSCLC patient, and high expression levels of ANKRD22 were significantly related to relapse and short OS time in NSCLC patients. In addition, ANKRD22 promoted cell proliferation and angiogenesis of NSCLC cells *in vitro* and *in vivo*, suggesting that ANKRD22 acts as an oncogene during the progression of NSCLC. Moreover, *in vivo* treatment with ANKRD22 siRNA decreased the size of xenograft tumors, suggesting that inhibition of ANKRD22 significantly inhibits tumor growth. Therefore, ANKRD22 is involved in the progression of NSCLC by regulating cell proliferation, and is a new biomarker or target for the diagnosis and therapy of NSCLC.

Ankyrin repeat proteins, which are usually described as being composed of linear arrays of tandem copies of the ankyrin motif, carry out a wide variety of biological activities and have been detected in various organisms ranging from viruses to humans^22^. The motif has now been recognized in >400 proteins, including cell cycle-related proteins, transcriptional regulators, cytoskeletal organizers, developmental regulators and toxins^23^. The number of ankyrin motif repeats within any one ankyrin repeat protein is highly variable, and ANKRD22 contains four ankyrin motif repeats. Although ANKRD22 has been demonstrated to be involved in the transition steps of the induced reprogramming process, the functions and underlying mechanisms of ANKRD22 are still largely unknown^21^. In this study, the potential function of ANKRD22 was investigated by gene knockdown coupled with microarray assay, and the genes influenced by ANKRD22 were enriched in the cell cycle pathway using bioinformatics analysis. Moreover, we also identified E2F1, which promoted cell cycle progression, as a target of ANKRD22 and found that ANKRD22 regulated the expression of E2F1 by activating the transcriptional activity of the E2F1 promoter. Moreover, ANKRD22 influenced cell proliferation, the cell cycle and E2F1 proliferative target genes by transcriptional regulation of E2F1. Therefore, it is suggested that ANKRD22 has a positive effect on the progression of NSCLC by transcriptional up-regulation of E2F1.

The role of the ankyrin motif in mediating protein-protein interactions has been well documented^24^. Unlike several other protein modules that recognize specific sequence motifs or protein modifications such as SH3 and SH2 domains, ankyrin repeat proteins do not bind selectively to a single class of protein targets^24^. Therefore, the diversity of the biological roles of ankyrin repeat proteins is paralleled by the diversity of unrelated proteins with which they interact. The Notch receptor is a key molecule in metazoan development, is involved in cell-cell signaling, and Notch has six ankyrin motif repeats which are very important in Notch function^25^. The IκB family constitutes a group of related molecules that act as inhibitors of the NF-κB transcription factors, and the structural domain of six ankyrins in IκB is essential for formation of the IκB/NF-κB complex^26^. The data presented here support the idea that ANKRD22 plays a role in the promotion of E2F1 transcription, and we speculated that the binding of RNA pol II on the E2F1 promoter may be influenced by ANKRD22. Although the mechanism underlying the regulation of E2F1 expression by ANKRD22 remains unclear, it is thought that ANKRD22 acts as a docking protein for multiprotein complexes that allows transcription of E2F1 to occur. Future investigations to determine the exact mechanism of transcriptional regulation by ANKRD22 and to identify other involved cofactors are warranted.

Uncontrolled cell proliferation is an invariable characteristic throughout the process of cancer development including initiation and progression^27^. E2F1 is a member of a large family of transcription factors containing conserved DNA binding domains that bind target promoters and regulate the expression of genes directly involved in the cell cycle, and E2F1 can promote cells through the G1/S checkpoint and acts as a positive regulator of cell proliferation^28^. Numerous studies have focused on the regulation of E2F1, and a long-standing paradigm is that E2F1 activity is tightly regulated by the retinoblastoma tumor suppressor (RB) which can inhibit the transcriptional activity of E2F1 by binding with E2F1^29^. Mutations of the RB that induce E2F1 activity and lead to unscheduled progression through the cell cycle have been identified in a number of human cancers^30, 31^. Recently, in addition to RB inactivation, overexpression and/or amplification of E2F1 has also been observed in NSCLC^32–34^. In this study, we also observed a significant upregulation of E2F1 in NSCLC primary carcinoma tissues compared with adjacent carcinoma tissues. Moreover, it has been demonstrated that tight control of E2F1 is crucial for tissue homeostasis and that overexpression of E2F1 may have a causative relationship with cancer development that extends beyond traditional control of the cell cycle, such as invasion and migration^35^. However, the molecular mechanism of deregulated E2F1 expression is poorly understood. Here, we found that ANKRD22 acts as a new and important transcriptional regulator to enhance E2F1 expression in NSCLC. Presumably, as successive up-regulation of ANKRD22 can occur during the progression of NSCLC, the overexpression of E2F1 can be promoted and maintained, and then NSCLC cells acquire uncontrolled proliferation and invasion features which finally result in metastasis and recurrence of NSCLC.

In summary, this is the first report to demonstrate that ANKRD22 exhibits oncogene activity that promotes tumor progression in NSCLC through the transcriptional regulation of E2F1. This study extends the knowledge regarding the regulation of E2F1 at the transcriptional level by ANKRD22, and suggests that ANKRD22 may be a potential novel therapeutic target for NSCLC. Such novel molecular mechanisms which sustain NSCLC have the potential to shed light on NSCLC and provide a molecular basis for tumor progression.

## Materials and Methods

### Ethics statement

All patients provided written informed consent, and the collection of NSCLC tissues for research purposes was approved by the relevant human research ethics committees of the Cancer Center of Guangzhou Medical University (Approval no. [2014] 100). All experiments were performed in accordance with relevant guidelines and regulations.

### Tissue sample collection

Forty-seven patients diagnosed with NSCLC were recruited from the Cancer Center of Guangzhou Medical University (Guangzhou, China) with informed consent and Institutional Review Board (IRB) permission, and the patient characteristics are shown in Table S6. Fresh samples of primary and adjacent tissues were obtained by surgery or aspiration biopsy. Snap-frozen samples were stored at −80 °C until use.

### Cell culture and transfections

Human NSCLC cells, H1299 and A549 cells were cultured in RPMI 1640 medium containing 10% fetal bovine serum at 37 °C. The expression plasmid of ANKRD22 was constructed by inserting cDNA into the pCDNA3.1 plasmid. The shRNA of ANKRD22 was designed for lentivirus production. The siRNA of E2F1 was purchased from the company. shRNA lentiviral vectors were transfected into cells by lentivirus, plasmids and siRNAs were transfected into cells by Lipofectamine 2000 (Invitrogen). Cells were harvested 48 or 72 hours after transfection for analysis.

### Microarray detection of mRNA expression profile

Total RNA in NSCLC cells and tissue samples was isolated using a Total RNA Purification Kit. RNA integrity was determined by 1% formaldehyde denaturing gel electrophoresis. RNA expression profile analysis was performed using the validated GeneChip Human Transcriptome Array 2.0 and PrimeView Human Gene Expression Array (Affymetrix). The RNA samples were hybridized on Affymetrix chip arrays, and the hybridization images were scanned by a laser scanner.

### RNAi screening of candidate genes using high content screening

The shRNAs of 10 screened genes were used for green fluorescent protein (GFP) labeled lentivirus production. H1299 and A549 cells were seeded in a 96-well plate, and each candidate shRNA lentivirus was added to the plate at the multiplicity of infection (MOI) of 0.3 with serum-free medium. Following infection of cells with different shRNA lentiviral vector, cell counting of GFP-positive cells was performed for five days by High Content Imaging Pathway Celigo (Nexcelom). The shRNA lentiviral vector expressing a scrambled sequence with no homology to the human genome was used as the negative control (shCtrl), and the shRNA lentiviral vector expressing a proto-oncogene specific-targeting shRNA for proliferation inhibition was used as the positive control (shPC).

### Xenograft tumors in nude mice

For the xenograft model assay, 1 × 10^7^ of H1299/ANKRD22-shRNA and H1299/control-shRNA cells were subcutaneously injected into 4 week-old BALB/c athymic nude mice, respectively. Each cell line was bilaterally injected into 6 mice, for a total of 12 injections. The longest diameter “L” and the shortest diameter “W” of tumors were measured five times in 30 days. The tumor volume was calculated using the following formula: tumor volume (mm^3^) = π/6 × L × W × W. After 30 days, all experimental mice were sacrificed simultaneously and tumor sizes were measured.

For the siRNA treatment assay, when the longest diameter of the xenograft tumor in nude mice reached 10 mm (approximately 20 days after injection of 1 × 10^7^ H1299 cells with stable luciferase expression), the mice were randomly divided into two subgroups (4 mice in each subgroup) and were treated with control siRNA (control group) or ANKRD22 siRNA (treatment group), respectively. To facilitate the introduction of siRNA into tumors, 200 μl of siRNA solution together with atelocollagen (30 μmol/L siRNA in 0.5% atelocollagen) was directly injected into each xenograft tumor. After 10 days of siRNA injections, D-Luciferin (15 mg/mL) was injected intraperitoneally into all experimental mice by 10 μl/g, and then all experimental mice were anesthetized and placed in the *in vivo* Bioluminescence Imaging to observe the fluorescence.

### Dual-Luciferase reporter assays

Luciferase reporter plasmids of the E2F1 promoter region (−483 to +25 bp) were constructed as pGL2-E2F1 plasmids. The pGL2-control plasmids were used as negative controls. Cells were transfected with 500 ng/well of pGL2 plasmid and 100 ng/well of pRL-TK using Lipofectamine (Invitrogen). After 24 hours, cells were transfected with the ANKRD22-plasmid. After another 48 hours, cells were harvested and analyzed for luciferase activity using the Dual Luciferase Reporter Assay (Promega) in a Clarity Luminescence Microplate Reader (BioTek). The relative luciferase activity (firefly luciferase) was normalized to pRL-TK activity (*Renilla* luciferase). Results were expressed as a fold induction over that of empty pGL2 activity.

### Chromatin immunoprecipitation (ChIP)

The ChIP Assay Kit (Millipore) was used for ChIP analysis. Chromatin DNA was extracted and broken into fragments of 200–400 bp in length by sonication. The chromatin fragments were then immunoprecipitated with the following antibodies: IgG and anti-RNA pol II (Abcam). Both the precipitated DNA fragments and the genomic DNA were used for RT-PCR. By using the same primer pairs, the intensity of the PCR products from the precipitated DNA fragments was normalized against the intensity of the PCR products of the genomic DNA. Primers specific for the E2F1 promoter region (−202 to +25 bp) are listed in Table S7.

### Real-time quantitative PCR (RT-PCR)

Total RNA in cells was extracted by Trizol (Invitrogen). For reverse transcription, cDNA was synthesized from 1 μg RNA by using Reverse Transcription Kit (Takara). Using GAPDH as internal control, RT-PCR was performed with the SYBR Green Realtime PCR Master Mix Kit (Toyobo) by using the ABI ViiATM7Dx Real-Time PCR System (Life Technologies). The mRNA RT-PCR primers are listed in Table S7.

### Western blotting

Cells were harvested and lysed using RIPA buffer for 0.5 hour at 4 °C. Fifty micrograms of proteins were loaded onto 15% SDS–PAGE for analysis. Rabbit polyclonal anti-ANKRD22, anti-E2F1, anti-CCNE2, anti-CDC6 and anti-GAPDH (internal control) primary antibodies (Cell Signaling, 1:1000 dilutions) were included and the cells were incubated overnight at 4 °C. Then, HRP (horseradish peroxidase) conjugate goat-anti-rabbit secondary antibody (Cell Signaling, 1:1000 dilution) was added and incubated for 4 hours. The bound antibodies were detected using the ECL Plus Western Blotting Detection system (GE Healthcare).

### Cell growth assay

Cell growth was determined using MTT (3-[4,5-dimethylthiazol-2-yl]-2,5 diphenyl tetrazolium bromide, Sigma). Briefly, NSCLC cells were transfected with ANKRD22-plasmid or ANKRD22-shRNA in 96-well plates. After 48 hours, the medium was removed and both 90 µl of RPMI-1640 medium (Gibco) and 10 µl of MTT solution were added to each well. After 2 hours incubation, the absorbance at 570 nm wavelength was measured on an automated reader (TECAN).

### Colony formation assay

NSCLC cells were transfected with ANKRD22-plasmid or ANKRD22-shRNA in a 60-mm Petri dish. After 48 hours, the cells were trypsinized and plated to assess clonogenic survival (1000 cells per well in six-well plates). Cells were allowed to form colonies over 7 days. The cells were then stained with GIEMSA and counted using ImageJ software.

### Cell apoptosis assay

Annexin V-FITC Apoptosis Detection Kit (BD) was used for cell apoptosis analysis. NSCLC cells were transfected with ANKRD22-plasmid or ANKRD22-shRNA in a 60-mm Petri dish. After 48 hours, cell apoptosis was determined by flow cytometry.

### Cell cycle assay

NSCLC cells were infected with ANKRD22-plasmid or ANKRD22-shRNA in a 60-mm Petri dish. After 48 hours, the cells were stained with propidium iodide (PI), and cell cycle was determined by flow cytometry.

### Statistical analysis

All microarray data were processed and normalized using the RMA algorithm and differential expression analysis was performed using Affymetrix software. Signal pathway enrichment analysis and cis-regulatory modules enrichment analysis were performed using Ingenuity Pathway Analysis. The Student’s unpaired t-test, chi-square test and Kaplan-Meier survival analysis were performed using SPSS 21.0 statistical software (IBM). A value of *P* < 0.05 (two tailed) was considered statistically significant.

## Electronic supplementary material

Supporting Information
