# Supplementary material for: ANKRD22 promotes progression of non-small cell lung cancer through transcriptional up-regulation of E2F1
Source: Sci Rep. 2017 Jun 30;7:4430. doi: 10.1038/s41598-017-04818-y (PMC5493668; doi:10.1038/s41598-017-04818-y)
Supplement: Supplementary file 1 — Supporting Information [file 41598_2017_4818_MOESM1_ESM.pdf]

ANKRD22 promotes progression of non-small cell lung cancer through transcriptional up-regulation of E2F1

Jun Yin, Wenfan Fu, Lu Dai, Zeyong Jiang, Hongying Liao, Wenbin Chen, Lei Pan, Jian Zhao*

Department of Chest Surgery, Affiliated Cancer Hospital & Institute of Guangzhou Medical University, Guangzhou 510095, Guangdong, China

*Corresponding author: tottijun@163.com

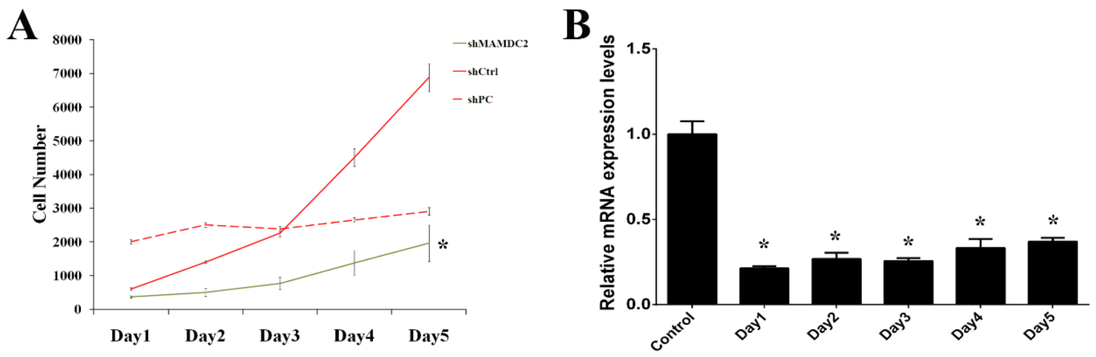

Figure S1. Effect of ANKRD22 on cell growth. **(A)** The cell growth curve (cell number) indicated that knockdown of ANKRD22 expression significantly inhibited H1299 cell growth. **(B)** The mRNA levels of ANKRD22 were decreased within 5 days after transfection with ANKRD22-shRNA in H1299 cells. (n=3, $*P < 0.05$)

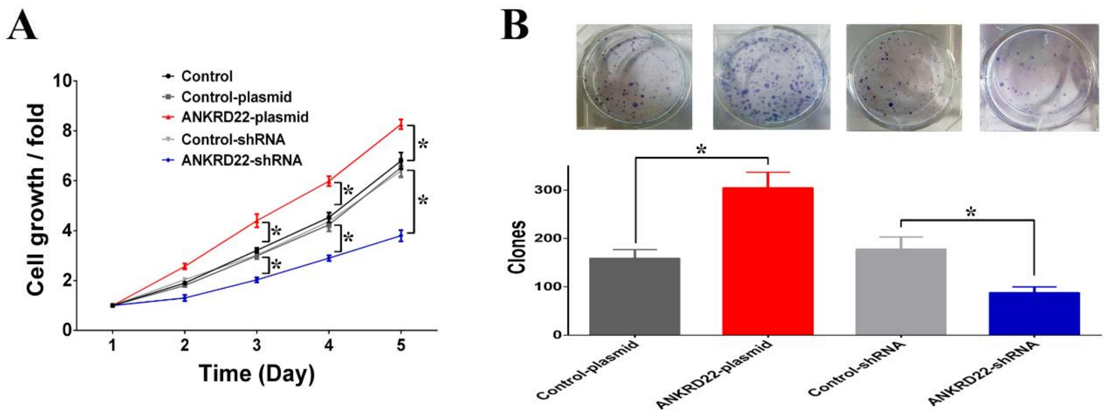

Figure S2. ANKRD22 regulated cell proliferation. **(A)** Overexpression of ANKRD22 increased cell growth and knockdown of ANKRD22 decreased cell growth in A549 cells. **(B)** Overexpression of ANKRD22 increased colony formation capacity and knockdown of ANKRD22 decreased colony formation capacity in A549 cells. (n=3, *$P < 0.05$)

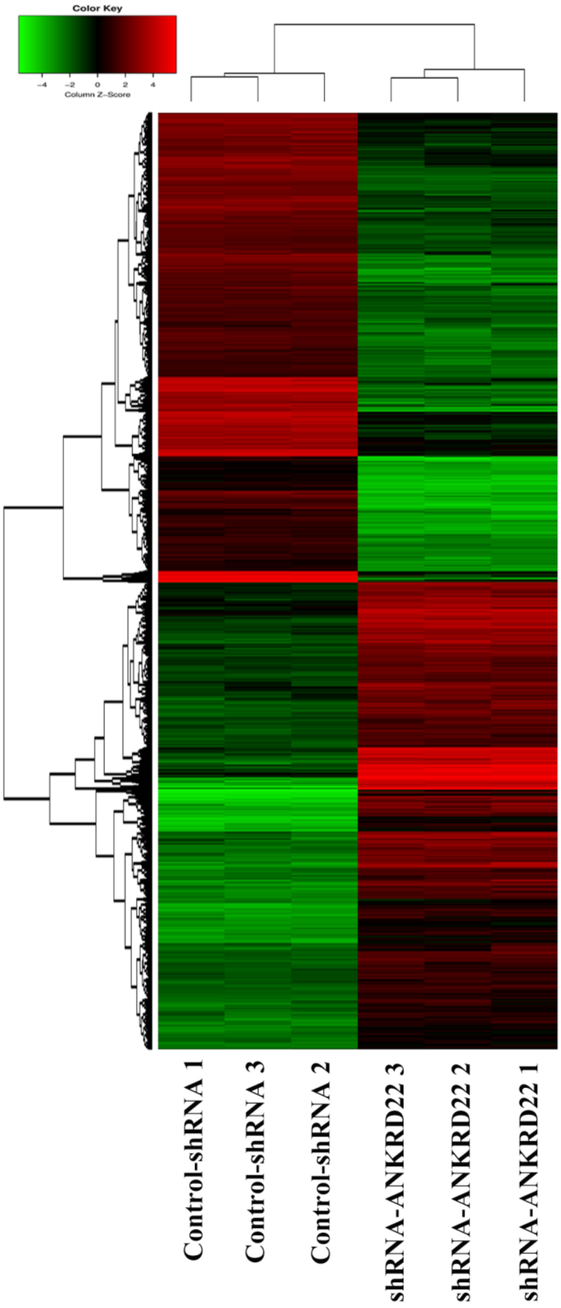

Figure S3. Clustering of differential genes in shRNA-ANKRD22 group compared with Control-shRNA group. Expression levels were depicted as color variation from red (high expression) to green (low expression) according to color bar scale.

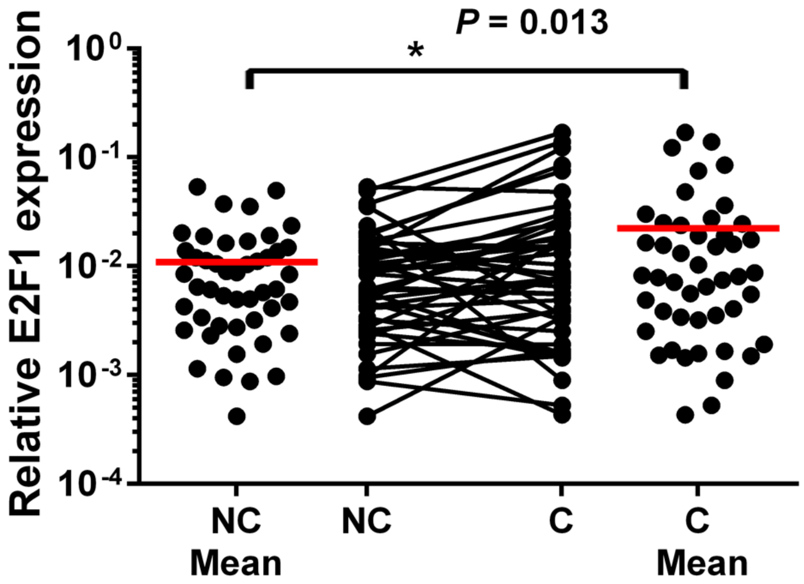

Figure S4. Scatter plots of expression levels of E2F1 in NC and C tissues. NC, adjacent carcinoma tissues; C, primary carcinoma tissues.

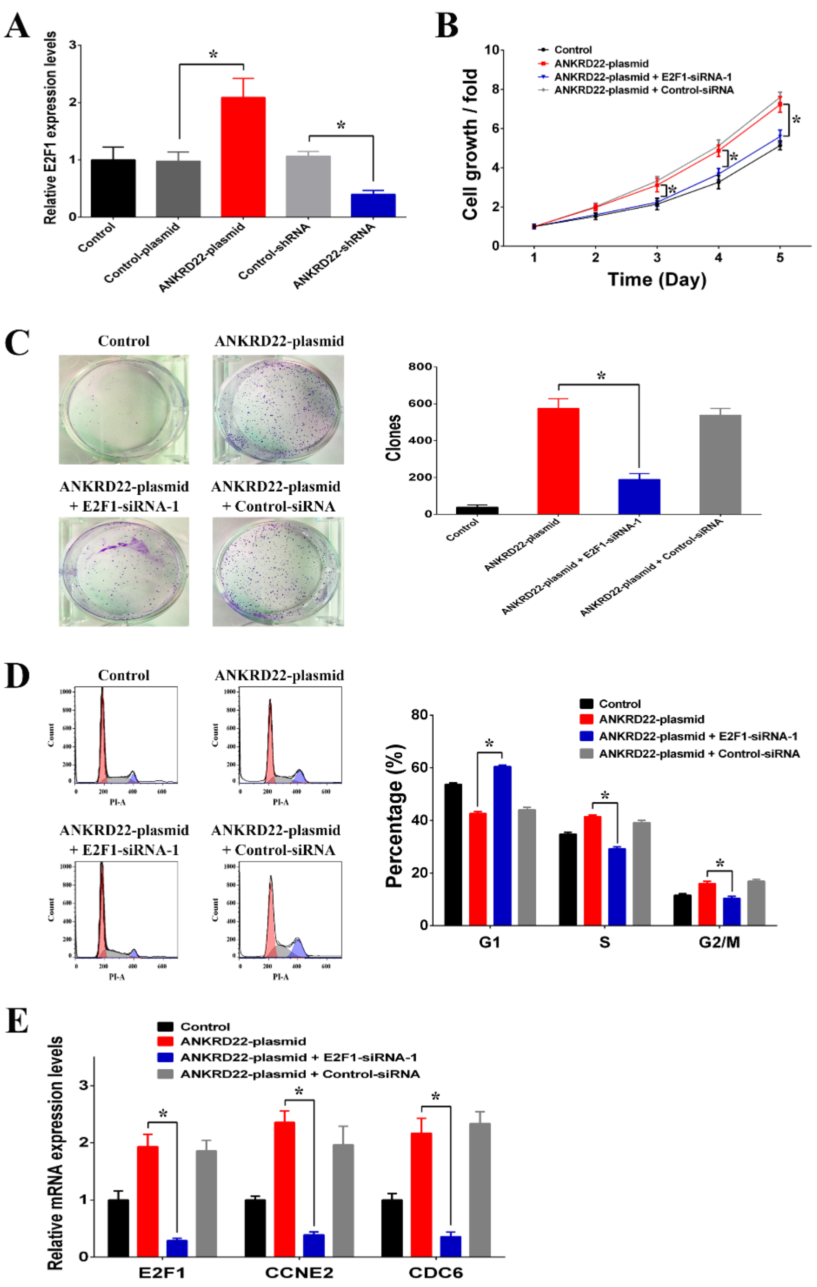

Figure S5. ANKRD22 regulated the cell cycle and cell proliferation by regulating E2F1. **(A)** Overexpression of ANKRD22 increased and knockdown of ANKRD22 decreased the mRNA expression levels of E2F1 in A549 cells. **(B)** Silencing of E2F1 reduced the promotion of cell growth by ANKRD22 in A549 cells. **(C)** Silencing of E2F1 reduced the promotion of colony formation capacity by ANKRD22 in A549 cells. **(D)** Silencing of E2F1 reduced promotion of the cell cycle by ANKRD22 in A549 cells. **(E)** In A549 cells, silencing of E2F1 reduced promotion of the expression of E2F1 target genes CCNE2 and CDC6 by ANKRD22. (n=3, *$P < 0.05$)

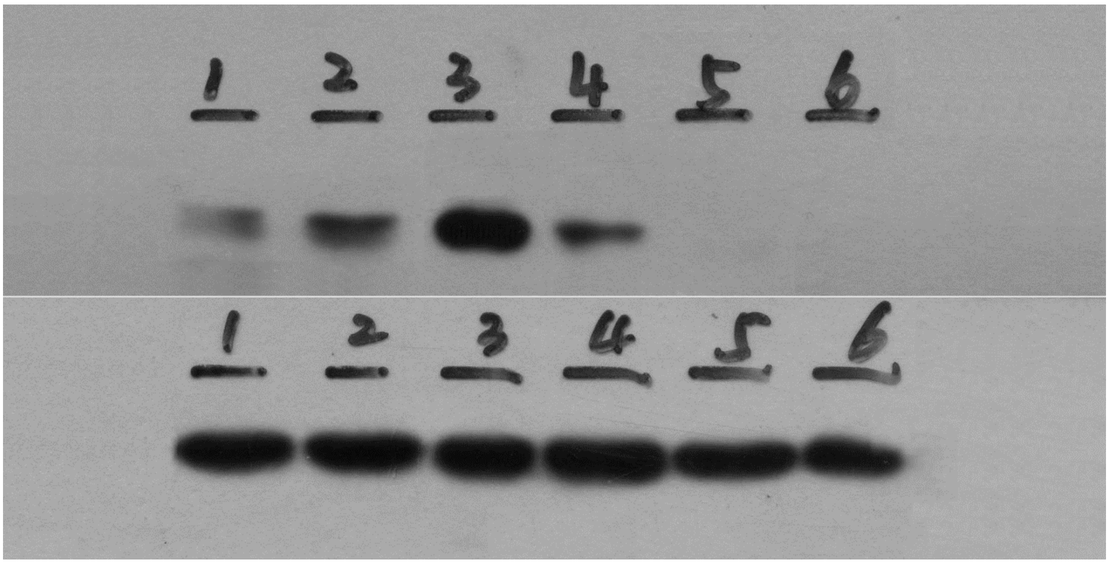

Figure S6. Full-length blots for Figure 4A

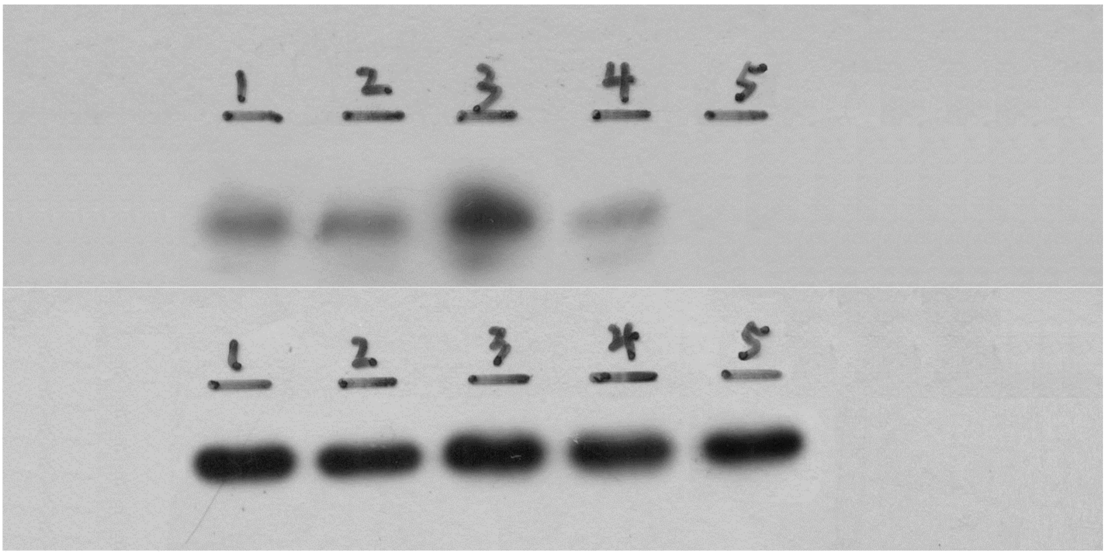

Figure S7. Full-length blots for Figure 6B

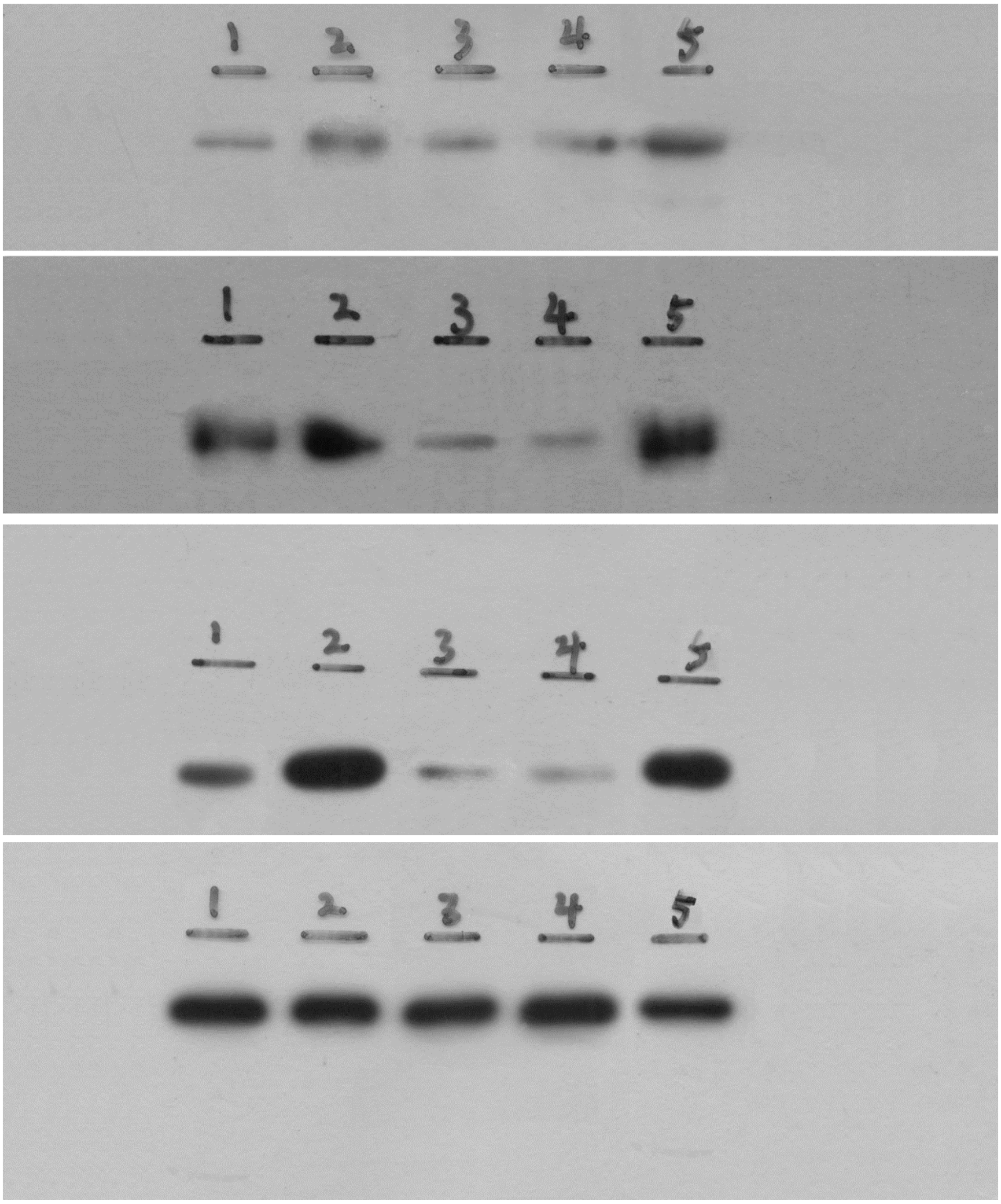

Figure S8. Full-length blots for Figure 7D

Table S1. Patient characteristics for microarray analysis.

Patient	Gender	Age	Histological type	Metastasis Sites
1	Male	71	Adenocarcinoma	Brain
2	Male	50	Adenocarcinoma	Brain
3	Female	72	Adenocarcinoma	Liver

Table S2. Cell count of H1299 cells transfected with ten candidate genes shRNAs, negative control shRNA (shCtrl) and positive control shRNA (shPC), respectively.

**Group**	**The average value of cell count**				
	**Day 1**	**Day 2**	**Day 3**	**Day 4**	**Day 5**
shCtrl	605	1402	2267	4505	6879
shPC	2003	2503	2389	2648	2908
shANKRD22	367	500	773	1381	1959
shMAMDC2	415	663	1003	1747	2614
shARNTL2	631	1322	2072	3814	5662
shEMBP1	210	406	579	1126	1966
shBAZ1A	270	536	807	1613	2830
shCKS2	467	974	1630	3189	4944
shGALNT1	492	1046	1763	3465	5466
shPLEKHH2	435	942	1596	3167	5039
shVEPH1	248	524	919	1889	2993
shLMCD1	273	583	942	1971	3327

Table S3. Correlation between expression levels of ANKRD22 and the clinicopathological features of NSCLC patients. (*P<0.05)

Clinicopathological factors		Expression levels[1] of ANKRD22			χ^2 value[2]	*P* value[2]
		**High**	**Medium**	**Low**		
**Histological type**	Adenocarcinoma	16	7	7	1.338	0.247
	Squamouscarcinoma	12	2	3		
**Gender**	Male	20	6	5	0.921	0.337
	Female	8	3	5		
**Age (years)**	<60	15	4	6	0.004	0.948
	≥60	13	5	4		
**Smoking**	Nonsmoker	14	4	8	0.794	0.373
	Smoker	14	5	2		
**TNM Clinical stage**	I	7	1	6	3.360	0.339
	II	8	5	2		
	III	6	2	2		
	IV	7	1	0		
**Metastasis**	0	21	8	10	**3.112**	**0.078**
	1	7	1	0		
**Relapse**	0	17	7	10	**4.692**	**0.030***
	1	11	2	0		

1. Expression levels of ANKRD22 were used the fold change between C and NC as categorical variables, where the categories represented as follows: cases with Fold Change ≥ 1.5 were designated as “High”, cases with 1.5 > Fold Change ≥ 1 were designated as “Medium”, whereas cases with Fold Change < 1 were designated as “Low”.

2. The chi-square test was performed between the group “High” and the sum of group “Medium” and “Low”.

Table S4. Results of Signal pathway enrichment analysis.

**Ingenuity Canonical Pathways**	**-log(*P* value)**	**Ratio**
Role of BRCA1 in DNA Damage Response	10.9	0.397
**Cell Cycle: G1/S Checkpoint Regulation**	10.3	0.422
**Role of CHK Proteins in Cell Cycle Checkpoint Control**	7.99	0.4
**Cell Cycle Control of Chromosomal Replication**	7.18	0.447
**Estrogen-mediated S-phase Entry**	6.85	0.542
**Cyclins and Cell Cycle Regulation**	6.74	0.321
Molecular Mechanisms of Cancer	5.12	0.179
Hereditary Breast Cancer Signaling	5.1	0.232
ATM Signaling	4.8	0.275
Protein Ubiquitination Pathway	4.72	0.192

Table S5. Differential genes related to pathway enrichment analysis.

**Ingenuity Canonical Pathways**	**Differential Genes**
Role of BRCA1 in DNA Damage Response	FAM175A,MRE11A,E2F3,BRCC3,RAD51,FANCE,FANCB,GADD45A,FANCD2,RFC2,BLM,BRCA1,STAT1,E2F2,CHEK2,ATM,E2F4,TOPBP1,FANCG,SMARCE1,MDC1,PLK1,MSH2,RFC4,SMARCA2,CDKN1A,E2F1,ATR,FANCA,RFC3,PHF10
Cell Cycle: G1/S Checkpoint Regulation	PA2G4,SMAD3,PAK1IP1,CDKN2C,E2F3,HDAC6,MYC,HDAC11,TGFB2,SMAD4,E2F2,ATM,CDC25A,CCNE2,E2F4,TFDP1,CDK6,MDM2,HDAC5,SKP2,E2F1,CDKN1A,TGFB3,GNL3,ATR,BTRC,CDKN1B
Role of CHK Proteins in Cell Cycle Checkpoint Control	E2F4,MDC1,MRE11A,PLK1,E2F3,PPP2R5A,CDK1,RAD1,PCNA,RFC4,CDKN1A,E2F1,RFC2,CLSPN,ATR,BRCA1,PPP2R1B,CHEK2,E2F2,RFC3,ATM,CDC25A
Cell Cycle Control of Chromosomal Replication	MCM5,CDC45,CDT1,POLA1,CDC6,ORC6,CDK6,DNA2,CDC7,MCM4,PCNA,MCM2,POLA2,DBF4,CHEK2,MCM7,ORC1
Estrogen-mediated S-phase Entry	E2F4,CCNE2,TFDP1,E2F3,CDK1,SKP2,MYC,CCNA2,CDKN1A,E2F1,CDKN1B,E2F2,CDC25A
Cyclins and Cell Cycle Regulation	CCNE2,E2F4,TFDP1,PA2G4,CDK6,CDKN2C,E2F3,CDK1,PPP2R5A,SKP2,HDAC5,HDAC6,CCNA2,HDAC11,CDKN1A,E2F1,TGFB3,TGFB2,ATR,BTRC,CDKN1B,PPP2R1B,E2F2,ATM,CDC25A
Molecular Mechanisms of Cancer	PRKACB,RAP2B,SMAD3,NCSTN,CDKN2C,MYC,LAMTOR3,ITGA3,BBC3,RHOB,PLCB1,NFKBIB,BRCA1,FRS2,BIRC3,FZD2,PRKD1,E2F2,CDC25A,ATM,RND2,E2F4,CCNE2,TFDP1,RALB,PTCH1,CDK6,PIK3R3,RHOQ,RABIF,E2F1,ARHGEF6,TGFB3,MAPK10,APH1B,ARHGEF18,FZD5,MAP2K3,ARHGEF9,PA2G4,BMPR2,E2F3,PRKCZ,FA

	NCD2,TGFB2,PRKCE,SMAD4,BID,CHEK2,BMP1,NAIP,PMAIP1,NRAS,SMAD6,MDM2,FOS,PLCB4,WNT10A,CDKN1A,PRKAG2,ATR,CYCS,CDKN1B,BMP6,JAK3,BCL2L11,PRKAR1A
Hereditary Breast Cancer Signaling	NPM1,MRE11A,HDAC6,RAD51,FANCE,FANCB,FANCD2,GADD45A,HDAC11,RFC2,BLM,BRCA1,FRS2,CHEK2,ATM,NRAS,FANCG,TUBG1,SMARCE1,CDK6,CDK1,HDAC5,PIK3R3,RFC4,MSH2,SMARCA2,H2AFX,CDKN1A,E2F1,ATR,FANCA,RFC3,PHF10
ATM Signaling	TOPBP1,MDC1,MDM2,MRE11A,CDK1,ATF2,SMC1A,RAD51,GADD45A,FANCD2,H2AFX,CDKN1A,MAPK10,BID,TP53BP1,ATR,HIST1H4J,BLM,BRCA1,CHEK2,ATM,CDC25A
Protein Ubiquitination Pathway	HSPA14,PSMB8,SACS,UBE2B,BAG1,UCHL5,USP40,BRCA1,BIRC3,UBE2Q1,USP38,USP9X,DNAJC2,UBE2S,THOP1,SKP2,HSPA8,UBE2G2,PSMD11,UBE2H,PSMB2,PSMD12,HSPA13,SMURF2,DNAJB6,UBE2C,UBE2I,B2M,CDC20,CDC23,UBE4A,USO1,DNAJC4,HSPE1,NEDD4,MED20,PSMD13,HSPH1,MDM2,USP30,UBE2L6,USP33,PSMD8,DNAJC11,DNAJC21,PSME1,USP37,BTRC,USP46

Table S6. Patient characteristics for clinical correlation analysis.

**Clinicopathological factors**	
**Total:**	
N	47
**Gender, n (%):**	
Male	31 (66.0%)
Female	16 (34.0%)
**Age, years (range):**	
Mean	57.9 (37-79)
**Histological type, n (%)**	
Adenocarcinoma	30 (63.8%)
Squamouscarcinoma	17 (36.2%)
**TNM Clinical stage, n (%)**	
I	14 (29.8%)
II	15 (31.9%)
III	10 (21.3%)
IV	8 (17.0%)

Table S7. Sequences of ChIP RT-PCR primers and RT-PCR primers.

**Primer**	**Sequence**
E2F1 promoter F	5′-CCTGGTACCATCCGGACAAA-3′
E2F1 promoter R	5′-AGTCCCGGCCACTTTTACG-3′
ANKRD22 F	5′-GACCCCACAATAAAGAATAAGC-3′
ANKRD22 R	5′-CCCACAGACCAAAAGTCTAAAA-3′
E2F1 F	5′-AGGCCCTCGACTACCACT-3′
E2F1 R	5′-CCAAGCCCTGTCAGAAAT-3′
CDC6 F	5′-AAGGGCGTTGGGGTCATAAG-3′
CDC6 R	5′-GGCTTCATCTAAGGGCAGCA-3′
CCNE2 F	5′-GGAACCACAGATGAGGTCCAT-3′
CCNE2 R	5′-CCATCAGTGACGTAAGCAAACT-3′
GAPDH F	5′-TGACTTCAACAGCGACACCCA-3′
GAPDH R	5′-CACCCTGTTGCTGTAGCCAAA-3′
